# Nebulized Heparin for Post-COVID-19-Related Hypoxia

**DOI:** 10.1055/s-0041-1732340

**Published:** 2021-07-30

**Authors:** Mehmet Hursitoglu, Erhan Eroz, Mehmet Akif Ozgul

**Affiliations:** 1 Internal Medicine Department, Basaksehir Cam & Sakura Sehir Hospital, University of Health Sciences, Istanbul, Turkey; 2 Pulmonary Medicine and Interventional Pulmonology Department, Basaksehir Cam and Sakura Sehir Hospital, University of Health Sciences, Istanbul, Turkey

To the editor

The pulmonary manifestations of coronavirus disease 2019 (COVID-19) may require a long time follow-up and a special therapeutic approach (such as pulmonary rehabilitation, supplementary oxygens, etc.). 1 2 3 This may end with pulmonary fibrosis that necessitates long time oxygen therapy. 4 Most of these patients are hospitalized because of oxygen supply need that exceeds the capacity of portable or home oxygen concentrator devices. 5 Nebulized and/or inhaled unfractionated heparin is investigated in some disease conditions. Some examples of these are smoke inhalation-related lung injuries, adult respiratory distress syndrome (ARDS), and pulmonary fibrosis. These trials were all safe and successful somewhat. 6 7 8 Here, we report two cases of post-COVID-19-related disabling respiratory distress conditions that were treated with nebulized unfractionated heparin administration.

The first case was that of an 81-year-old female COVID-19 patient. She was admitted to our hospital's intensive care unit (ICU) for mechanical ventilation support. A three day pulse 250 mg and 80 mg maintenance dose of IV methylprednisolone was started. Also, she had received diuretics and different antibiotics. After 24 days of ICU unit care, she has been transmitted to our medical world with a 10 L O _2_ supply using a non-rebreather (NRB) facemask. Her ICU maintenance treatment of subcutaneous low molecular weight heparin (LMWH) *enoxaparin* sodium 0.6 mL twice daily, methylprednisolone 40 mg PO daily continued. During her seventh day of follow-up at our medical ward, *N* -acetylcysteine 300 mg IV thrice daily was also initiated. Despite these maintenance therapies, her O _2_ need was not decreased. On the 14th day of medical ward follow-up, still, her O _2_ requirement was 8 to 10 L (to keep pulse oxymetry oxygen saturation [sO _2_ ] at 92%). The ground glass appearances, bronchial dilatations, and cardiomegaly are evident in her chest CT ( Fig. 1 ). Her insistence on home discharge was continued even at this critical level of O _2_ supply need. After a thorough search for possible nonharmful therapeutic approaches at these conditions, the suggestion of using nebulized heparin was made by the first author. In addition to the COVID-19 inpatient written consent form, this treatment was discussed with the patient and her first-degree kin. After a positive response, the first dose of 10,000 IU UFH (Koparin [Kocak Farma Ltd. Co.]) was administered by nebulizer for 1 hour (diluted in 3 mL of 0.9% NaCl solution). At the next day of this treatment, her oxygen need was decreased to 5 L (sO _2_ was 92%) (without any noted side effects). So, the dose of nebulized UFH increased to twice daily thereafter. At the end of third day of nebulized UFH treatment, her sO _2_ became 94% (with only a 4 L nasal O _2_ supply). Because of her insistence, she was discharged home with a portable O _2_ concentrator treatment support. On the 10th day of discharge, she has contacted by phone. She claimed that she is doing well. Her sO _2_ is approximately 95% (with a 3–4 L O _2_ supply) ( Table 1 for the progress).

**Table 1:** Pre- and post-nebulized unfractionated heparin (UFH) treatment days cases' oxygen saturation and need

Days		−1	0	1	2	3	4	5	10
Nebulized UFH	*Case 1*	*−*	*+*	*+*	*+*	*+*	*−*	*−*	*−*
	Case 2	−	+	+	+	+	+	−	−
O _2_ need (L)	*Case 1*	*8–10*	*8–10*	*5*	*5*	*4*	−	−	*3–4*
	Case 2	10	10	8	8	8	8	7–8	4
sO _2_ (%)	*Case 1*	*92*	*92*	*92*	*94*	*94*			*94*
	Case 2	90–91	90	93	93	93	92–93	93	94

**Fig. 1:**
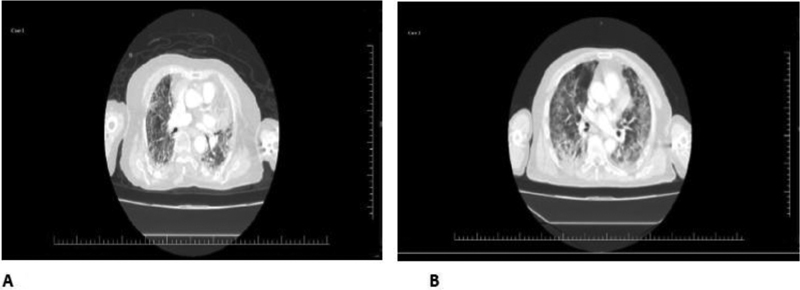
Views from chest computed tomography of the cases ( **A** = Case1, and **B** = Case 2).

The second case was that of a 67-year-old male COVID-19 patient. His chest CT shows consolidations, ground-glass appearance, and bronchial dilations ( Fig. 1 ). On the 17th day of admission, NRB facemask O _2_ support was not successful in keeping his sO _2_ levels above 92%. So, heated and humidified high-flow nasal oxygen support was started. Supportive maintenance steroid and LMWH were all given. On the 29th day of hospital admission, O _2_ needs were settled at 10 L (with sO _2_ levels of 90–91%). With the courage of positive results of our nebulized UFH therapy in the above first case, we discussed this treatment option with this patient too. After a positive response, as in the above previous patient, this UFH treatment was initiated as 10,000 IU twice daily. On the day of starting this treatment, the sO _2_ level was 91% (with a 10 L NRB facemask O _2_ supply). The day after starting this therapy, his sO _2_ was raised to 93% (with an 8 L O _2_ supply). As in the first case, this patient was discharged on the fifth day of completion of UFH therapy with a 2 to 3 L O _2_ supply need only (sO _2_ = 94%) ( Table 1 ).

## Discussion

Autopsy studies in COVID-19 showed that the rate of pulmonary fibrosis increase with the duration of the disease in ARDS patients. These fibrosis detection rates were 4, 24, and 61% in <1 week, 1 to 3 weeks, and >3 weeks, respectively. The pulmonary route of administration of heparin is tried in some types of respiratory diseases and non-COVID-19 related ARDS conditions before. There are no reports of major (or even minor) even with a maximum 120,000 IU unfractionated heparin per day. 6 7 8 The main reason for the long-time hospital stay of our first case was the high need for O _2_ (10 L). So, after informing the patient about nebulized UFH trials, she accepted our suggestion. On first day, only a single dose of UFH 10,000 IU was tried. The next day, a dramatic decrease in O _2_ need was observed. So, a twice daily 10,000 IU of nebulized heparin was continued later. In our patients series, the absolute eosinophil count of severe critically ill COVID-19 patients is so low (mostly zero) ( *unpublished data* ). Experimental studies showed that heparin inhibits allergen-induced eosinophil infiltration into the lung by a mechanism other than its anticoagulant activity. 9 As we know, cytotoxic eosinophil granule proteins are implicated in the pathogenesis of some airway diseases. The protective activity of heparin may be related to its local effect of neutralization of eosinophil cationic protein. 10 This may explain partially the obvious effect of nebulized UFH on improving sO _2_ levels of both cases that the parenteral heparin failed to show such effect. But we should mention that heparin has antiviral, anti-inflammatory, and mucolytic effects as well. 11 This easy and cheap seemly effective treatment model needs to be pointed out in future studies. Its safety and usefulness were also confirmed at the early phase of COVID-19 infection. 12 Using such cheap and safe drugs during such challenging pandemic and a global risk of economic crises is of paramount importance (even in developed countries). So, it seems that nebulized UFH treatment is safe and effective in reducing O _2_ need at long-standing COVID-19-related hypoxia. Still, further studies are needed to confirm this.
